# Rapid and long‐lasting remission of refractory Hailey‐Hailey disease by IL‐13 inhibition with tralokinumab

**DOI:** 10.1111/ddg.70023x

**Published:** 2026-01-12

**Authors:** Oliver Brandt, Stephanie M. Huber, Simon M. Mueller

**Affiliations:** ^1^ Dermatologische Klinik Universitätsspital Basel Basel Schweiz

**Keywords:** Dupilumab, Interleukin‐13, Morbus Hailey‐Hailey, Pemphigus chronicus benignus familiaris, Tralokinumab

Dear Editors,

Hailey‐Hailey disease (HHD; OMIM 169600), also known as pemphigus chronicus benignus familiaris, is a rare, autosomal dominant, chronic genodermatosis that usually manifests for the first time in early adulthood with complete penetrance but variable expressivity. It affects the intertriginous areas, especially the axillae, groin and, in women, the submammary regions, where erosive, macerated, fissured, erythematous plaques develop. The associated pain and frequent foetor often lead to social isolation, which further impairs the quality of life of patients.^1^ Mechanical irritation, sweating, UV exposure and microbial and viral infections can aggravate the disease or can trigger a relapse.

The disease is caused by a mutation in the calcium pump‐encoding *ATP2C1* gene that leads to disturbances in intracellular calcium homeostasis and thus to impaired suprabasal keratinocyte adhesion and acantholysis.^2^

We report on a 68‐year‐old female patient with a positive family history (father and both uncle and grandmother on the father's side were affected) who had suffered from histologically confirmed Hailey‐Hailey disease for 38 years, and who did not respond or only poorly responded to numerous therapy attempts but became symptom‐free under treatment with the anti‐interleukin (IL)‐13 antibody tralokinumab.

The skin lesions had previously persisted in the axillae, submammary and inguinal areas (Figure 1a, b) and regularly worsened during the warm season due to sweating, causing the patient to wear her underwear inside out to avoid additional mechanical irritation from seams. In addition, during periods of deterioration, she refrained from sports activities such as Nordic walking, swimming and cycling, as well as activities that caused friction in the affected areas of her body. Recurrent infections in these areas aggravated the foetor, resulting in increased social isolation of the patient.

**FIGURE 1:**
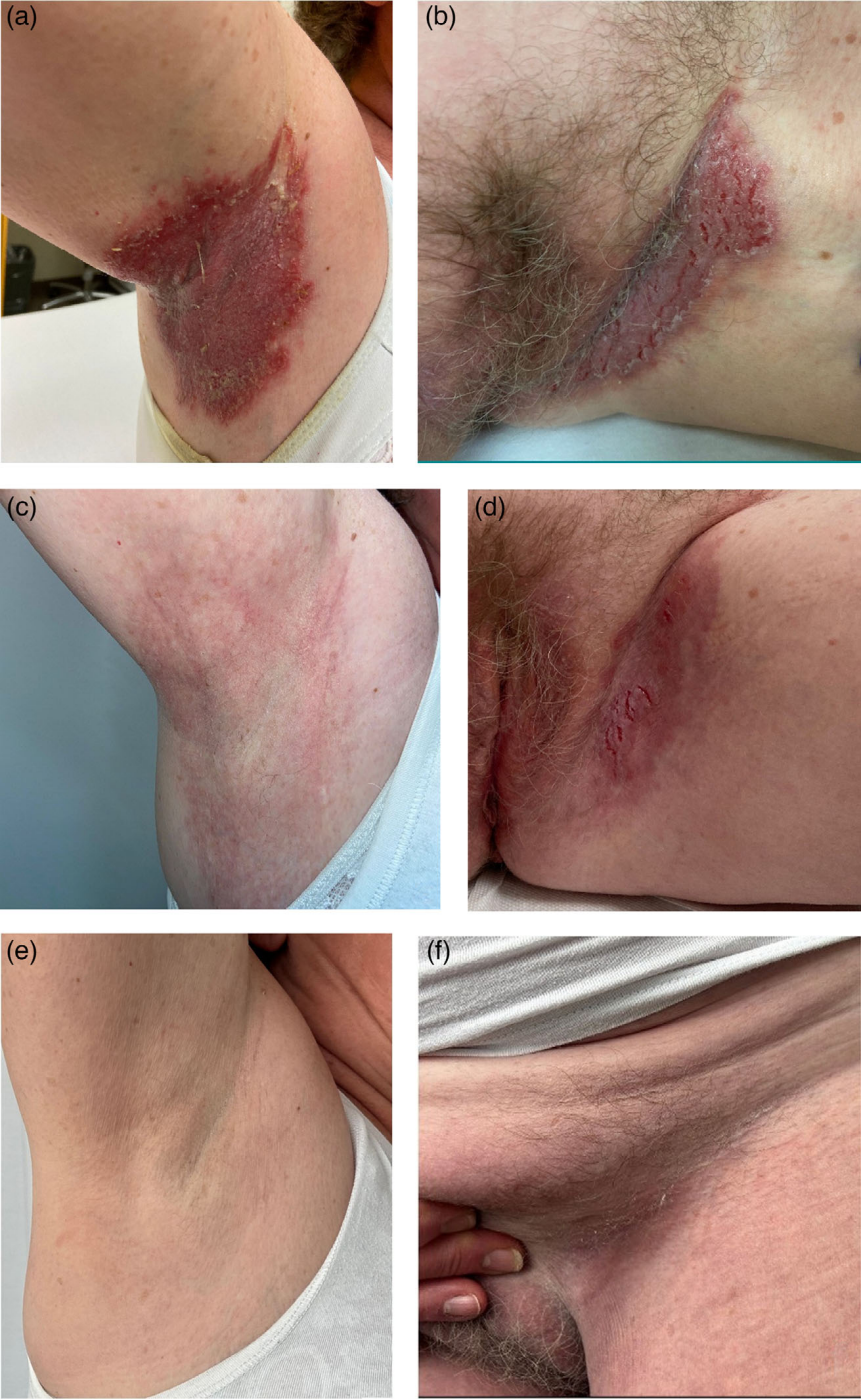
Hailey‐Hailey disease lesions at baseline and after treatment with tralokinumab. (a, b) Erosive, erythematous‐livid, macerated plaques interspersed with fissures in the axillary and inguinal regions before starting treatment, (c, d) after eight weeks of treatment, and (e, f) five months after the last application of tralokinumab 300 mg.

Treatments with systemic and topical corticosteroids, neotigason, isotretinoin, antibiotics and CO_2_ laser therapies, each in combination with once‐daily octenidine dressings, led to only marginal improvement at best or had to be discontinued prematurely due to adverse side effects.

After the skin condition had again deteriorated significantly at the end of 2023 and the patient had to be admitted to hospital due to severe erosions and intense pain, we initiated an off‐label therapy with the IL‐13 inhibitor tralokinumab, initially at a dose of 600 mg, which was subsequently continued with 300 mg every 14 days in combination with the previously administered octenidine dressings once daily.

Within just a few weeks after the first application, the lesions had improved significantly and the patient was considerably less affected by the disease (Figure 1c, d), which was also reflected in a decrease in the Dermatology Life Quality Index (DLQI) from an initial score of 26/30 to 20/30 points. Except for a brief exacerbation after a three‐week holiday in Italy in midsummer, the skin lesions continued to heal. Therefore, treatment with tralokinumab and antiseptic local therapy was discontinued on a trial basis in October 2024 after six weeks without symptoms.Therefore, treatment with tralokinumab and antiseptic local therapy was discontinued on a trial basis in October 2024 after six weeks without symptoms. The patient has now been symptom‐free for eleven months (Figure 1e, f), practices Nordic walking regularly, and enjoys swimming and cycling. Treatment of the formerly affected areas of the body with over‐the‐counter skin care creams is only carried out after sporting activities; the latest DLQI score was 1/30 points.

Until recently, treatment of HHD was difficult, with significant and long‐lasting improvements rarely achieved.^3^ In the last few years, several reports have been published on the efficacy of dupilumab in HHD, an antibody targeting the IL‐4 receptor, which among other conditions is approved for the treatment of atopic dermatitis (AD).^4^, ^5^ The authors observed a significant improvement or even healing of the lesions within a few weeks, without any notable adverse side effects. Similar findings were published by Garg et al. who, like us, also describe a rapid onset of action with excellent tolerability of tralokinumab in a recently published case report.^6^ The fact that both antibodies have been shown to be highly effective in the treatment of HHD implies that type 2 inflammatory processes are crucial to the pathophysiology of the disease. However, the exact mechanisms of action are not yet known.

In recent years, the prominent role that IL‐13 plays not only in AD but also in other types of chronic inflammation in the skin has been recognized.^7^ The cytokine is also expressed after barrier damage by type 2 innate lymphoid cells (ILC2s) and, above all, by Th2 cells that they recruit via keratinocyte alarmins. It is therefore conceivable that the oxidative stress reaction induced in keratinocytes by disturbed Ca^2+^ homeostasis triggers an inflammatory response associated with barrier damage which is then exacerbated and perpetuated by IL‐13.

Furthermore, in vitro experiments have shown that IL‐4 and IL‐13, both individually and in combination, influence Ca^2+^ mobilization in keratinocytes and inhibit their differentiation,^9^ as well as reduce the expression of structural and adhesion molecules such as desmoglein1 and desmocollin1. The latter could be the cause of the frequently detectable acantholysis in HHD.^10^ Inhibition of IL‐13 by tralokinumab or dupilumab could thus explain their excellent efficacy in HHD.

Our observation that selective inhibition of IL‐13 is sufficient for effective treatment of HHD and may even be disease‐modifying suggests that this cytokine, as in atopic dermatitis, is instrumental in maintaining the chronic inflammatory process and therefore plays a significant role in the pathogenesis of HHD. Future studies will have to show whether drugs that inhibit the inflammatory response mediated by IL‐13 can become a standard of care in the treatment of HHD.

## CONFLICT OF INTEREST STATEMENT

Oliver Brandt has received grants for congress attendance from the following pharmaceutical companies or has acted as a consultant for them: Abbvie, Bayer, Beiersdorf, Jansen/ Johnson & Johnson, Leo Pharma, Novartis, Pfizer, Sanofi, UCB. Stephanie M. Huber has received grants for congress attendance from Merz Pharma Schweiz and Stallergenes. Simon Müller declares that he has received honoraria and/or funding in the last three years for consultancy and/or speaker engagements and/or as a clinical trial investigator for the following companies: Sanofi‐Aventis AG, Galderma SA, Janssen‐Cilag AG, LEO Pharmaceutical Products Sarath, medtis GmbH, Amgen, Incyte, La Fonderie SAS, Novartis Pharma AG.
